# Prevalence and Predictors of Self-Reported Adverse Experiences in Digital Meditation Training: 2 Randomized Controlled Trials

**DOI:** 10.2196/90602

**Published:** 2026-06-12

**Authors:** Polina Beloborodova, Lillian M Smith, Kevin M Riordan, Otto Simonsson, Lilah T Dottori, Helen Q Song, Nicholas S Shashko, Raquel Tatar, Scott A Baldwin, Amit Bernstein, John D Dunne, Richard J Davidson, Matthew J Hirshberg, Simon B Goldberg

**Affiliations:** 1 Center for Healthy Minds, University of Wisconsin–Madison, 625 W. Washington Ave, Madison, WI, 53703, United States, 1 608-263-6321; 2 Department of Counseling Psychology, University of Wisconsin–Madison, Madison, WI, United States; 3 Center for Social Sustainability, Department of Neurobiology, Care Sciences and Society, Karolinska Institutet, Solna, Stockholm County, Sweden; 4 Humin, Madison, WI, United States; 5 Department of Psychology, Brigham Young University, Provo, UT, United States; 6 Department of Asian Languages and Cultures, University of Wisconsin–Madison, Madison, WI, United States; 7 Department of Psychology, University of Wisconsin–Madison, Madison, WI, United States; 8 Department of Psychiatry, School of Medicine and Public Health, University of Wisconsin–Madison, Madison, WI, United States

**Keywords:** meditation, adverse effects, adverse experiences, well-being, digital interventions

## Abstract

**Background:**

Digital meditation-based interventions (MBIs) reach vast global audiences with millions of active users, yet concerns persist about the frequency and nature of adverse experiences (ie, AExs) occurring during meditation training. Some researchers have argued that AExs are substantially underdetected and reflect iatrogenic harm caused by meditation (ie, adverse effects [AEfs]). Others contend that these experiences largely reflect common stressors that would be experienced without meditation. These competing perspectives underscore the need for further research, particularly in the context of digital MBIs, the most widely used form of meditation training.

**Objective:**

This study examined the prevalence, predictors, and subjective evaluations of AExs during a digital MBI and tested whether reported experiences may be caused by meditation practice via comparisons between meditation-exposed and nonexposed participants.

**Methods:**

Data were drawn from 2 trials of the Healthy Minds Program. Exploratory study 1 (n=315) consisted of a sample of distressed US undergraduate students to estimate the prevalence of AExs and identify baseline predictors. Preregistered confirmatory study 2 (n=594) sampled distressed US adults from all 50 states to replicate findings from study 1 and to examine participants’ subjective evaluations of AExs. Study 2 additionally compared AEx rates between participants who did and did not complete guided meditations to assess whether AExs could be caused by meditation exposure. Study 3 (n=87) used qualitative methods to analyze study 1 participants’ responses to an open-ended question regarding their strategies for coping with AExs.

**Results:**

In studies 1 and 2, 27.9% (88/315) and 10.1% (40/396) of participants, respectively, reported at least one AEx during the study period, with 6.7% (21/315) and 3% (12/396) reporting functional impairment, largely aligning with previous research. Critically, in study 2, rates of AExs did not significantly differ between participants who did and did not complete guided meditations, suggesting that these experiences were not caused by meditation practice. Higher baseline depression, anxiety, loneliness, experiential avoidance, and perceived barriers to meditation predicted more frequent AExs. In studies 1 and 2, 89.8% (79/88) and 90% (36/40) of participants who reported AExs, respectively, indicated that they were glad to have learned to meditate. Qualitative analyses showed that participants used diverse coping strategies, often using skills learned through the Healthy Minds Program.

**Conclusions:**

AExs were relatively common but occurred at comparable rates among participants who did and did not meditate, challenging claims that such experiences were caused by meditation practice in distressed individuals. Although a small subset of participants reported some degree of functional impairment, most evaluated their AExs as tolerable and described their overall MBI experience as positive. Together, these findings highlight the importance of distinguishing AExs that likely reflect epiphenomena of preexisting distress or symptoms from iatrogenic harm attributable to MBIs.

## Introduction

Meditation-based interventions (MBIs) have become widely recognized treatments for a range of mental and physical health conditions, with comparable efficacy to established psychological treatments [1-4]. A growing literature has investigated distressing experiences occurring in the context of meditation practice [5-8]. Historically, contemplative traditions themselves acknowledged that a range of distressing experiences (ie, adverse experiences [AExs]) can occur during meditation training (eg, Zen sickness [9]) and distinguished between experiences interpreted as salutary progress in practice and those reflecting iatrogenic processes that warrant intervention (ie, adverse effects [AEfs]) [9]. The research community’s attention to these phenomena was further prompted by influential qualitative studies documenting substantial disruption and impairment, often occurring in the context of intensive meditation practice [10].

More recently, several studies have quantitatively investigated the occurrence of distressing experiences during meditation interventions. For example, results from large-scale cross-sectional studies of meditators suggest that distressing experiences (eg, anxiety and reexperiencing traumatic memories) attributed to meditation practice are reported by 50% to 78% of the participants [11,12]. Distressing experiences have also been studied in the context of standardized MBIs such as mindfulness-based cognitive therapy, with 39% to 58% of the participants reporting them [6,13]. These data points highlight that, contrary to prototypical popular press portrayals of meditation as peaceful and pleasant [14], many individuals attribute a host of distressing experiences to their practice of meditation.

Distressing experiences in digital meditation interventions may be particularly important to investigate. These programs now reach large global audiences, with the top 10 meditation apps together reaching over 300 million downloads [15]. Meditation apps are by far the most widely used type of mental health app [16]. Recently, 3 randomized controlled trials (RCTs) testing digital meditation training, 2 delivered via a smartphone app and the third combining synchronous and asynchronous digital components, evaluated intervention-related harm, operationalized as depression and anxiety symptom worsening. Merits of these studies included relatively large to large sample sizes (from n=343 to n=2315) and randomized controlled designs that included a waitlist control comparison group [17-19]. The comparison to waitlist control is critical here, as the waitlist received no intervention and therefore provides an estimate of worsening symptoms in the absence of a digital meditation intervention. In all 3 studies, random assignment to the meditation condition resulted in a significantly lower likelihood of symptom worsening relative to waitlist. In other words, assignment to the meditation condition was protective against symptom worsening. An important limitation of these studies is that they did not purposively measure distressing experiences occurring during the intervention period, which may be underreported if they are not purposively sampled [6,8].

As digital meditation programs are widely used, claims about their potential to produce distress or harm have important public health implications. Monitoring of symptom worsening also contributes to the body of work understanding AExs in mental health digital interventions broadly, where negative consequences have been documented across a range of app types and clinical populations; however, many clinical trials do not collect such data and therefore the picture is still emerging [20]. Careful measurement of these experiences and equally careful interpretation of the resulting data are essential. It is clear that AExs are relatively common in the context of meditation training [5,6]; however, it is not clear that meditation causes these experiences or that these experiences are indicators of iatrogenic harm. Many previous studies have made implicit or explicit causal claims (ie, AEfs) while relying on uncontrolled designs and retrospective self-report wherein participants subjectively attribute distressing experiences to meditation [7]. These methods are relatively weak methodological approaches for establishing causal inferences [21-23]. To exercise greater care with causal claims, we distinguish between AExs, meaning distressing or aversive experiences that occur in the context of meditation, and AEfs, negative experiences or consequences *caused* by meditation. In this nomenclature, the word “effect” connotes strong evidence for causality.

Studying who might be at risk for AExs in the context of digital meditation training merits systematic investigation because these programs are usually implemented without instructor supervision and guidance. The unsupervised nature of digital MBIs may increase vulnerability to AExs. In in-person MBI trials, participants with greater baseline psychological vulnerability (eg, adverse childhood experiences [24] and elevated baseline mental health symptoms [25]) report higher rates of AExs. Meditation trains sustained attention to present-moment experience; for individuals with elevated vulnerability, this experiential field may include distressing thoughts, emotions, or trauma-related material [24]. Although longer-term mental training presumably increases the practitioner’s ability to regulate such experience by, for example, experiencing them with acceptance or nonjudgment [26], for novice practitioners, it is possible that increased awareness is not matched by the ability to accept the content of experience, precipitating AEx. Increasing the salience and accessibility of such content may precipitate AExs. Consistent with social baseline theory, which postulates that the human brain evolved to assume the availability of social resources and therefore regulates effort, emotion, and threat processing based on the presence of others [27], social support may serve as a protective factor, while the lack thereof could serve as another vulnerability factor [25].

Another area that has not been sufficiently investigated is meditation practitioners’ subjective evaluations of challenging and distressing experiences. Some definitions of AExs include experiences that meditation traditions would regard as part of the normal phenomenology of becoming familiar with the mind, such as increased awareness of one’s difficult emotions [9]. Relatedly, it is possible that even when an AEx is reported on a scale item, the practitioner may not see it as negative or unproductive [8,9]. Even in the context of intensive meditation training that appears to have the greatest potential to elicit AExs [10], a quasi-experimental trial showed that meditation retreat participants reported peak AExs as being more salutary than adverse [28].

Taken together, differing perspectives on AExs and AEfs within meditation training exist in the literature [8,29], highlighting the need for further empirical work on this topic, especially in digital meditation training, the most widely used form of meditation training. These efforts can guide the responsible use of meditation apps—carefully evaluating their safety while simultaneously not overstating their danger or withholding an intervention approach that may produce benefits and protect against symptom worsening.

This research addresses gaps in the prior literature regarding the prevalence, predictors, causality, and appraisal of AExs in digital meditation training. The primary aims of the study were to assess symptom worsening and self-reported AExs that participants subjectively attribute to meditation, using measures designed for this purpose [30]. The secondary aim was to measure participants’ subjective appraisals of reported AExs, the coping strategies they used to manage these AExs, and their global appraisals of having learned to meditate. Additionally, we take a first step toward evaluating whether some of the reported AExs are caused by digital meditation training and can be classified as AEfs by comparing the rates of AEx between groups of participants who did and did not complete guided meditations.

Study 1 (n=315) was an exploratory secondary data analysis using a sample of distressed US undergraduate students. The parent study 1 trial was preregistered at ClinicalTrials.gov NCT04741529 and through the Open Science Framework (OSF) Registries [31,32]. This was followed by a preregistered confirmatory secondary data analysis (study 2, n=396) that aimed to replicate and extend the findings from study 1 with a sample of distressed US adults drawn from all 50 states. Study 2 hypotheses and data analyses were registered through the OSF Registries [33]. The parent study 2 trial was preregistered at ClinicalTrials.gov NCT06282523 and through the OSF Registries [34]. Finally, to gain insight into the strategies that participants used to cope with AExs, we conducted study 3 (n=87), a qualitative analysis of open-ended responses collected in study 1. In each study, participants used the Healthy Minds Program (HMP), a meditation app that combines guided meditations with brief psychoeducational podcast-style lessons on the science of well-being [35]. HMP consists of 4 modules focused on core well-being skills: attentional control and mindful awareness (awareness module), social connectedness and prosocial behavior (connection module), insight into self-concept and mental habits (insight module), and the identification and enactment of personal values and motivations (purpose module). The program has demonstrated efficacy in reducing psychological distress and preventing symptom worsening in 2 prior RCTs (n=343 [17] and n=662 [18]). Although the primary aim of both parent trials was to compare different versions of the HMP, all participants received the same core content, including guided meditations and didactic material. In study 2, a subset of participants completed the didactic lessons without engaging in guided meditations, which made it possible to assess whether participants who did not meditate reported AExs attributed to meditation. All materials, data, analysis code, and supplemental materials associated with this paper can be accessed at the OSF repository [36]. We report how we determined our sample size, all data exclusions, all manipulations, and all measures in the study. All participants in all studies provided their written informed consent before participating.

## Study 1

### Study 1 Overview

The aims of study 1 were threefold: (1) to estimate the prevalence of self-reported AExs attributed to meditation and symptom worsening during the HMP, (2) to assess how participants felt about having practiced meditation despite AExs, and (3) to examine potential predictors of AExs. Prior literature indicates that psychological vulnerability factors, such as baseline mental health symptoms and loneliness, are positively associated with the reporting of AExs [11,25]. Accordingly, we expected that psychological resilience factors would predict fewer AExs. Thus, we selected predictors representing psychological vulnerability and resilience from the available measures. Baseline levels on predictors are of particular interest, as most research on the topic is cross-sectional. As such, past reports of AEx frequency may include distressing experiences not directly caused by meditation training but instead reflecting an individual’s preexisting propensities toward challenging emotional states. Thus, predictors included demographic variables, baseline psychological vulnerability factors (baseline depression, anxiety, loneliness, experiential avoidance, and fear of missing out), baseline resilience factors (self-compassion), and meditation practice variables (amount of HMP app usage and perceived barriers to meditation).

### Methods

#### Ethical Considerations

All methods and procedures were reviewed and approved by the University of Wisconsin–Madison Institutional Review Board (protocol 2020‐0197) and carried out in accordance with the provisions of the World Medical Association Declaration of Helsinki. All participants provided their written informed consent electronically via REDCap (Research Electronic Data Capture) software [37] before participating. They earned US $55 for completing all study activities, including US $15 for completing 80% of the meditations, and had a chance to win a US $200 lottery prize. Participants were able to opt out of the study at any time without penalty. Identifying information was collected for compensation purposes only and kept confidentially on a secure university server. The data used in this study were deidentified before sharing.

#### Participants and Procedures

**Figure 1.**
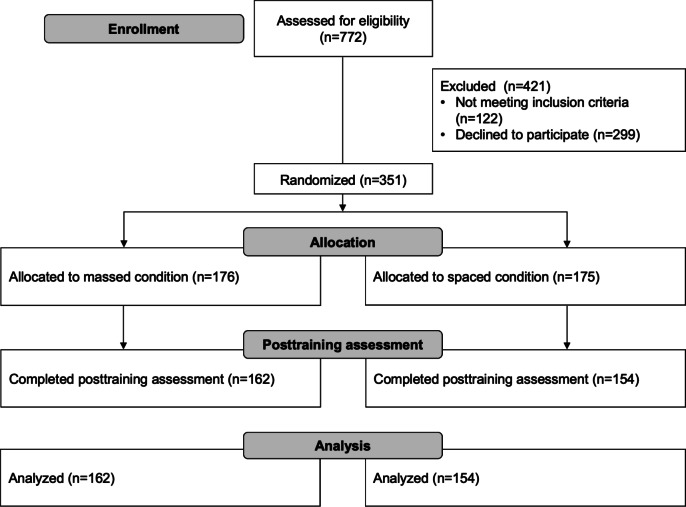
CONSORT (Consolidated Standards of Reporting Trials) diagram.

The study took place between March and April 2021. Undergraduate students were recruited via email from a large public university in the Midwestern United States. To be eligible, participants had to be aged at least 18 years, enrolled as undergraduate students, have access to a smartphone or compatible device (Android or iOS), and report elevated symptoms of anxiety and/or depression, defined as *T*-scores ≥55 on the Patient-Reported Outcomes Measurement Information System (PROMIS) Depression and/or PROMIS Anxiety short forms 4a [38]. Individuals with significant prior meditation experience—defined as having attended a meditation retreat, practicing weekly for more than 1 year, practicing daily for the past 6 months, or having received formal instruction beyond an introductory course—were excluded. These thresholds were chosen to minimize prior exposure to meditation and ensure that the study tested intervention effects among meditation-naïve participants. Participants with severe depression (*T*-score >70 on the PROMIS Depression short form) were also excluded. The baseline sample included 351 participants; 315 (89.7%) of them completed the postintervention AEx measures, and 316 (90%) completed depression and anxiety symptom measures. Figure 1 presents the CONSORT (Consolidated Standards of Reporting Trials) diagram. Participant demographics are presented in Table S1 at the OSF repository [36].

The primary aim of this trial was to compare different meditation schedules for their use of the HMP: the “spaced” condition, which involved two 10-minute sessions per day, or the “massed” condition, which involved one 20-minute session per day. There was no difference in psychological distress or other outcomes between conditions (see [39]). In the present analyses, group assignment was included as a control variable. Data were collected via the REDCap software at baseline and posttest assessment (ie, 2 weeks later). Participants were incentivized to complete daily practices.

#### Measures: Predictors

Demographic characteristics included age, gender identity, and racial and ethnic identity (see Table S1 at the OSF repository [36] for full demographic information). For statistical modeling, gender was categorized as woman versus not woman, and race was categorized as White versus non-White due to low frequency of other gender and race categories. HMP app usage was calculated as the number of days participants used the app and total minutes of meditation based on objective app logs.

In addition to demographic characteristics and HMP usage statistics, we tested the following hypothesized baseline predictors of AExs. Depression and anxiety were assessed with the 4-item PROMIS Anxiety (sample Cronbach α=0.80; McDonald ω=0.84) and 4-item PROMIS Depression Scales version 1.0, Short Form (sample α=0.88; ω=0.90) [38], scaled as *T*-scores using established US population means and SDs. Loneliness was evaluated with the 5-item National Institute of Health Toolbox Loneliness scale (sample α=0.89; ω=0.92) [40]. Experiential avoidance, the tendency to suppress or avoid unwanted internal experiences, was assessed using the 7-item Acceptance and Avoidance Questionnaire–II (sample α=0.89; *ω*=0.93) [41]. Fear of missing out (ie, anxiety that others are having rewarding experiences from which one is excluded) was measured with the 10-item Fear of Missing Out Scale (sample α=0.86; ω=0.91) [42]. Self-compassion (ie, treating oneself with kindness, understanding, and acceptance) was assessed with the 12-item Self-Compassion Scale Short Form (sample α=0.82; ω=0.86) [43]. Perceived barriers to meditation, including perceived lack of benefit, inadequate knowledge, pragmatic barriers, and sociocultural conflict, were evaluated with the 12-item Determinants of Meditation Practice Inventory-Revised (sample α=0.76; ω=0.81) [44]. Descriptive statistics of these measures are presented in Table S2 at the OSF repository [36].

#### Measure: AEx Outcomes

We planned to test 6 operationalizations of AExs (Table 1). First, the frequency of general self-reported AExs subjectively attributed to meditation was measured with the single item, “I had challenging, difficult, or distressing experiences as a result of my meditation during this study” that participants evaluated on a scale ranging from 1 (*never*) to 5 (*frequently*) [30]. Second, meditation-attributed impairment was measured among all participants with the item, “My meditation-related challenging, difficult, or distressing experiences impaired my ability to function,” rated on a scale ranging from 1 (*not at all*) to 4 (*severely*). This was followed by the item, “How long did your impairment last?” to measure the duration, with answer choices from 1 (1 *day or less*) to 4 (*longer than 1 month*) [30]. Notably, this metric of AEx was not tested in analyses due to insufficient frequency of self-reported impairment. Third, specific self-reported AExs, subjectively attributed to meditation, were measured with the Meditation-Related Adverse Effects Scale, Mindfulness-Based Program version [30] (MRAES-MBP). Participants were asked, “Did any of the following occur AS A RESULT OF MEDITATION during this study?,” followed by a list of 10 items, such as “I had trouble thinking clearly and/or making decisions,” rated on a scale ranging from 1 (*never*) to 5 (*longer than 1 month*), with an additional “other” item where the participants could describe their experience. The frequency of most response options was low (<10%); therefore, items were dichotomized for analysis (ie, occurrence vs no occurrence). Fourth, the count of reported AExs was computed across the 10 items. Our fifth and sixth operationalizations examined whether participants experienced a clinically significant symptom deterioration—worsening of depression and anxiety symptoms from baseline to posttraining, defined as a 3 *T*-score point increase, a lower bound of the minimal clinically important difference (MCID) range [45,46].

**Table 1.** Adverse experience outcomes.

Outcome^a^	Definition
Frequency of general AEx^b^ occurrence	The frequency of general self-reported AExs subjectively attributed to meditation by the study participants
Functional impairment	Functional impairment subjectively attributed to meditation by the study participants
Reporting at least one specific MRAES-MBP^c^ AEx	Endorsing at least one item from MRAES-MBP [30]
Specific MRAES-MBP AEx count	Count of endorsed items from the MRAES-MBP
Depression symptom worsening	Clinically significant deterioration of depression symptoms from baseline to postintervention
Anxiety symptom worsening	Clinically significant deterioration of anxiety symptoms from baseline to postintervention

a These operationalizations were chosen to capture both subjective attributions and symptom changes, with the 3-point minimal clinically important difference as a conservative threshold.

b AEx: adverse experience.

c MRAES-MBP: Meditation-Related Adverse Effects Scale, Mindfulness-Based Program.

To probe participants’ subjective evaluations of their experience, we added the item, “Consider the experiences you had practicing meditation during this study, including any challenging, difficult, or distressing experiences. How much do you agree with the following statement: I am glad I have practiced meditation.” Participants evaluated it on a scale ranging from 1 (*strongly disagree*) to 6 (*strongly agree*) [11].

#### Statistical Analysis

We used different statistical approaches to analyze predictors of meditation-related AEx measures listed in Table 1. Functional impairment was not included as an outcome due to the small number of participants reporting it (n=18), which limited statistical power. Frequency of AEx occurrence was modeled using cumulative link models (also known as proportional odds models), which estimate the odds of moving from one Likert scale point to the next (eg, shift from “never” to “rarely” or higher) [47]. The likelihood of reporting at least one specific AEx and depression or anxiety symptom worsening were modeled using logistic regression. The count of reported specific AExs was analyzed using a zero-inflated negative binomial model, which accounts for excess zeros and skewness in the nonzero portion of the distribution [48].

We examined predictors of each outcome in a series of 9 models, which included demographic covariates (age, gender, and race and ethnicity) and condition (massed or spaced) along with one of the additional nondemographic predictors: depression, anxiety, loneliness, experiential avoidance, fear of missing out, self-compassion, HMP app usage, or perceived barriers to meditation. All additional predictors were standardized to allow comparison of effect sizes. Each model with an additional predictor was compared to a baseline model containing only demographic variables and condition using the deviance test for binary outcomes and the likelihood ratio test for continuous and ordinal outcomes. To account for multiple comparisons, false discovery rate (FDR) correction [49] was applied to *P* values for each outcome across all models. Demographic variables and condition were treated as substantive predictors in the baseline model, and their *P* values were included in the FDR correction set. However, in subsequent models, they were treated as covariates, and their *P* values were excluded from the correction set. Additionally, we compared AExs between participants who did and did not experience symptom improvement, defined as a 3 *T*-score point decrease in depression and anxiety symptoms (MCID [45,46]). We used the Mann-Whitney *U* test to compare the average frequency of AEx occurrence and the average number of specific AExs from the MRAES-MBP between these participant groups, with effect size calculated as the rank-biserial correlation. To compare the proportions of participants reporting at least one specific AEx from the MRAES-MBP, we used a 2-proportion z test, with effect size calculated as Cohen *h*. All analyses were conducted in R (version 4.4.0; R Core Team) with the packages *ordinal* [50] for the cumulative link models and *glmmTMB* [51] for the zero-inflated negative binomial models.

### Sample Size Justification

This study is a secondary analysis of data from a randomized trial whose sample size was determined by feasibility and powered to detect small-to-moderate between-group differences (*d*=0.30) on its primary outcome, as described in the original trial protocol [39]. While the current analysis was not specified before data collection, sensitivity power analysis with the *pwr* R package [52] showed that the available sample (n=315 participants who completed posttest AEx measures) was powered to detect zero-order correlations between hypothesized AEx predictors and AEx occurrence of *r*≥0.16 at 80% power.

### Results

#### Prevalence of AExs

**Figure 2.**
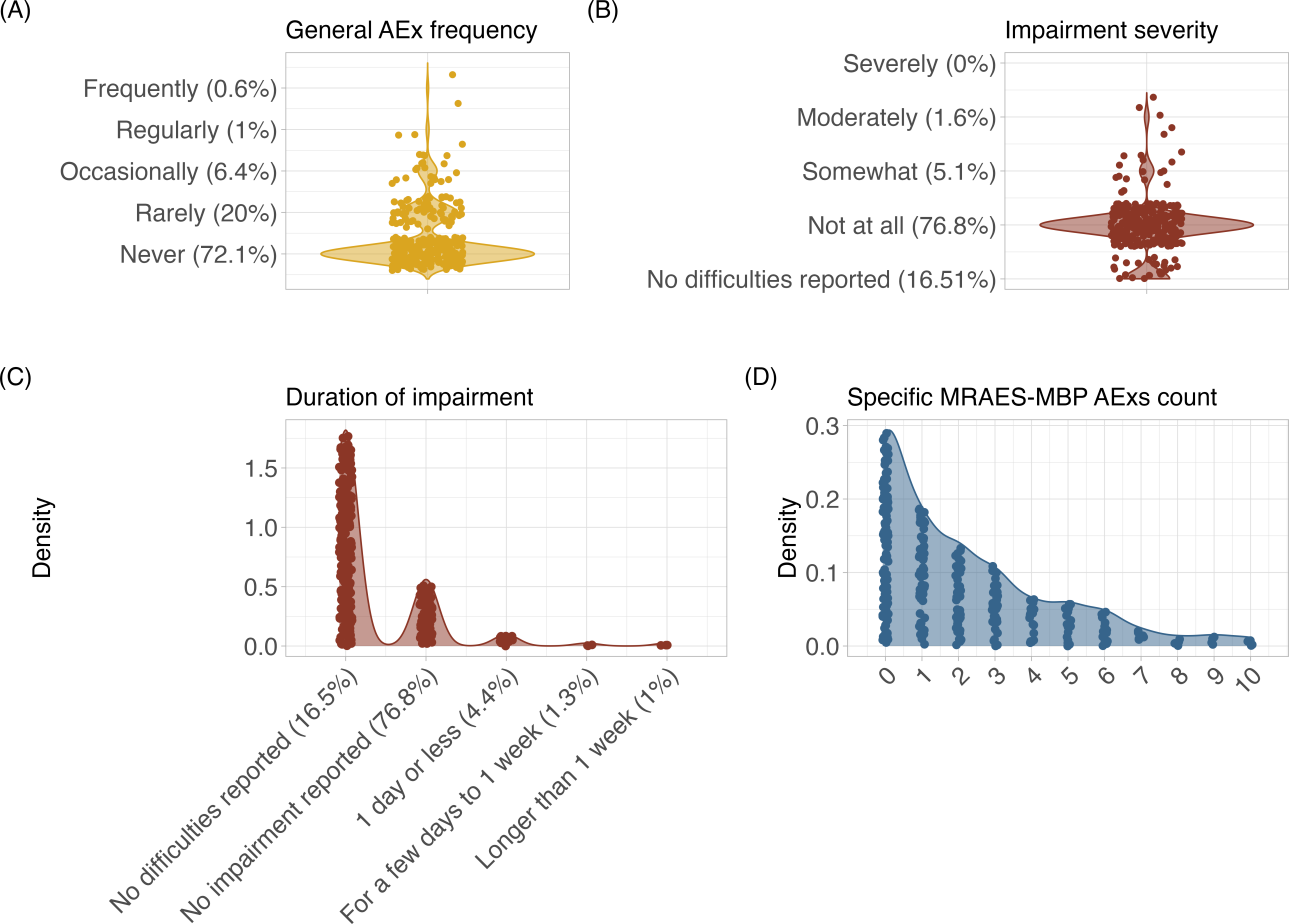
Prevalence of adverse experiences. Adverse experience (AEx) frequency refers to how often participants had challenging, difficult, or distressing experiences that they attributed to meditation during the study; impairment refers to impaired ability to function; specific Meditation-Related Adverse Effects Scale, Mindfulness-Based Program version (MRAES-MBP) [30] . AEx count refers to the number of endorsed experiences from the MRAES-MBP (out of 10).

As shown in Figure 2A, the majority of participants (227/315, 72.1%) did not report any challenging, difficult, or distressing experiences during the study. In addition, 20% (63/315) of participants reported experiencing them rarely, 6.4% (20/315) reported experiencing them occasionally, and 1.6% (5/315) reported experiencing them regularly or frequently. Furthermore, as depicted in Figure 2B, 93.3% (294/315) of participants reported no impairment, whereas 6.7% (21/315) reported impairment they attributed to meditation, with 5.1% (16/315) indicating that AExs they attributed to meditation somewhat impaired their ability to function, while 1.6% (5/315) reported moderate impairment. No participants reported severe impairment. Among the 6.7% (21/315) who reported impairment, the majority (14/315, 4.4% of the full sample) reported it lasting 1 day or less, while 1.3% (4/315) of the full sample experienced it for a few days to 1 week, and 1% (3/315) of the full sample experienced it for more than 1 week (Figure 2C).

When asked about specific AExs from the MRAES-MBP, 65.4% (206/315) of participants reported experiencing at least one. On average, participants reported 2.15 (SD 2.42) specific AExs, although the distribution was positively skewed (Figure 2D), indicating that while experiencing a low number of specific AExs was common, participants rarely endorsed the majority of them. Anxiety and reexperiencing stressful experiences from the past were the most frequently reported AExs, while anhedonia (“I had trouble enjoying things that I used to enjoy”) was the least frequently reported. The distributions of specific AExs are shown in Figure S1 at the OSF repository [35].

**Figure 3.**
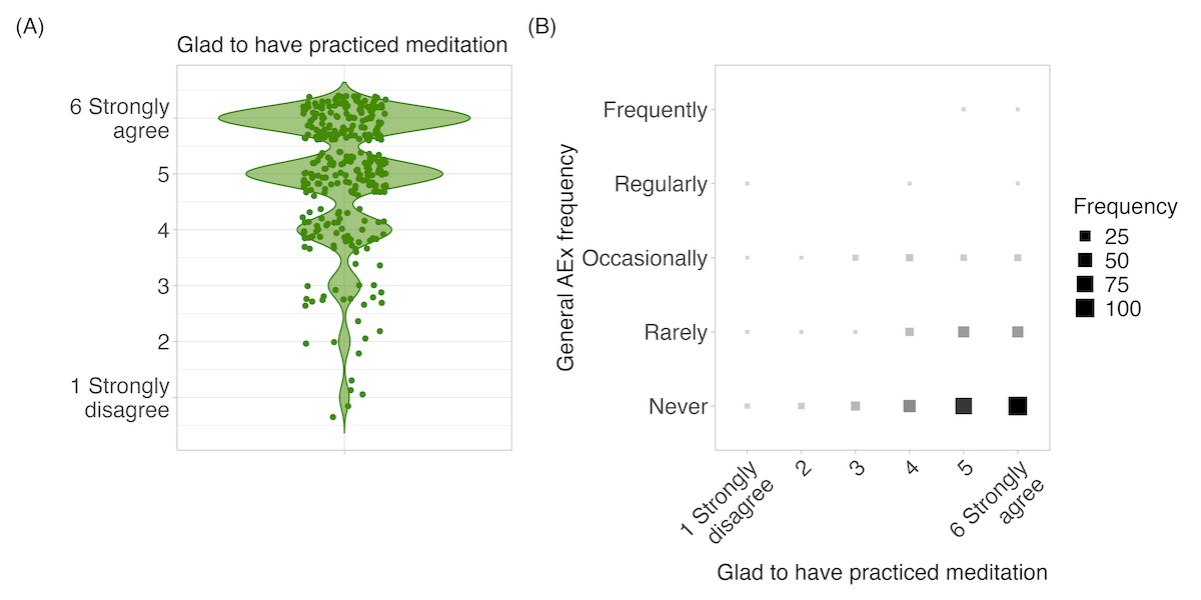
Glad to have practiced meditation. Adverse experience (AEx) frequency refers to how often participants had challenging, difficult, or distressing experiences that they attributed to meditation during the study.

The majority of participants reported being glad they had practiced meditation, including 91.6% (208/227) of those who did not experience any AExs (ie, selected “never” on the AEx frequency item) and 89.8% (79/88) of those who did report experiencing an AEx (ie, selected any other response), as shown in Figure 3.

Some participants experienced a clinically significant worsening of their depression (34/316, 10.8%) or anxiety symptoms (38/316, 12%), defined as a ≥3 *T*-score point increase from pre- to post-intervention on the respective measures [45,46]. To contextualize these findings, 51.9% (164/316) and 45.6% (144/316) of the participants experienced a clinically significant improvement defined as a ≥3 *T*-score point decrease in depression and anxiety symptoms, respectively, in the same period. None of the AEx measures differed between participants who did and did not experience improvement in depressive symptoms (*P* values >.53). Similarly, the number of specific AExs reported on the MRAES-MBP and the likelihood of reporting at least one specific AEx did not differ between participants who did and did not experience improvement in anxiety symptoms (*P* values >.61). Participants whose anxiety symptoms improved reported slightly more frequent AExs (mean 0.47, SD 0.77) than those whose symptoms did not improve (mean 0.27, SD 0.61), although the effect size was small (*r*=0.13; *P*=.008).

#### Predictors of AExs

This section summarizes the results of analyses testing predictors of AEx outcomes. Tables 2-4 summarize the model estimates and their statistical significance; full results are provided in Tables S3–8 at the OSF repository [36].

**Table 2.** Study 1: predictors of general adverse experience (AEx) count and reporting at least one specific MRAES-MBP^a^ AEx^b^.

Model and predictor	General AEx frequency			At least one specific MRAES-MBP AEx		
	OR^c^ (95% CI)		*P* value (FDR^d^)	OR (95% CI)		*P* value (FDR)
Model 1						
Age	1.18 (1.00-1.38)		.05	0.94 (0.81-1.09)		.48
Race: White	1.45 (0.81-2.69)		.26	0.71 (0.40-1.23)		.35
Gender: female	0.87 (0.52-1.49)		.70	0.77 (0.45-1.29)		.40
Group: spaced	1.04 (0.63-1.72)		.87	1.34 (0.83-2.16)		.35
Model 2						
Baseline depression	2.40 (1.78-3.33)		<.001	1.47 (1.16-1.89)		.007
Model 3						
Baseline anxiety	2.13 (1.61-2.87)		<.001	1.79 (1.39-2.35)		<.001
Model 4						
Baseline loneliness	1.70 (1.31-2.24)		<.001	1.39 (1.09, 1.78)		.02
Model 5						
Baseline experiential avoidance	2.49 (1.85-3.42)		<.001	1.80 (1.40-2.34)		<.001
Model 6						
Baseline fear of missing out	1.04 (0.80-1.34)		.82	1.15 (0.90-1.48)		.37
Model 7						
Baseline self-compassion	0.72 (0.55-0.93)		.02	0.86 (0.68-1.10)		.35
Model 8						
Minutes of HMP^e^ practice	0.98 (0.87-1.11)		.81	1.02 (0.91-1.14)		.83
Days of HMP usage	0.98 (0.80-1.21)		.87	0.99 (0.81-1.21)		.93
Model 9						
Baseline barriers to meditation	1.23 (0.95-1.60)		.14	1.42 (1.11-1.83)		.02

a MRAES-MBP: Meditation-Related Adverse Effects Scale, Mindfulness-Based Program version [30].

b Some estimates had CIs excluding the null value (OR 1) but did not remain statistically significant after FDR correction; original *P* values are omitted for brevity and provided in Tables S3-S8 at the OSF repository [36]; demographic variables and group are included in models 2‐9 as control variables, but their parameter estimates are omitted for brevity.

c OR: odds ratio.

d FDR: false discovery rate.

e HMP: Healthy Minds Program.

**Table 3.** Study 1: predictors of MRAES-MBP^a^ adverse experience (AEx) count^b^.

Model and predictor	MRAES-MBP AEx count					
	Negative binomial submodel			Zero-inflation submodel		
	β^c^ (95% CI)		*P* value (FDR^d^)	OR^e^ (95% CI)		*P* value (FDR)
Model 1						
Age	−0.04 (−0.12 to 0.04)		.61	1.05 (0.85 to 1.29)		.81
Race: White	−0.32 (−0.60 to −0.04)		.08	1.11 (0.45 to 2.73)		.92
Gender: female	−0.07 (−0.33 to 0.20)		.81	1.36 (0.56 to 3.33)		.76
Group: spaced	−0.16 (−0.42 to 0.10)		.53	0.50 (0.19 to 1.30)		.38
Model 2						
Baseline depression	0.10 (−0.03 to 0.23)		.35	0.58 (0.37 to 0.90)		.06
Model 3						
Baseline anxiety	0.07 (−0.06 to 0.20)		.60	0.42 (0.24 to 0.73)		.02
Model 4						
Baseline loneliness	−0.01 (−0.14 to 0.12)		.92	0.52 (0.30 to 0.90)		.07
Model 5						
Baseline experiential avoidance	0.13 (0.00 to 0.27)		.15	0.43 (0.23 to 0.82)		.046
Model 6						
Baseline fear of missing out	−0.01 (−0.15 to 0.12)		.92	0.78 (0.48 to 1.27)		.60
Model 7						
Baseline self-compassion	−0.06 (−0.20 to 0.09)		.75	1.21 (0.72 to 2.04)		.75
Model 8						
Minutes of HMP^f^ practice	−0.02 (−0.08 to 0.05)		.78	0.94 (0.77 to 1.15)		.78
Days of HMP usage	0.02 (−0.09 to 0.13)		.92	1.05 (0.76 to 1.46)		.92
Model 9						
Baseline barriers to meditation	−0.01 (−0.16 to 0.13)		.92	0.46 (0.25 to 0.85)		.06

a MRAES-MBP: Meditation-Related Adverse Effects Scale, Mindfulness-Based Program version [30].

b Some estimates had CIs excluding the null value (1 for OR and 0 for regression coefficients) but did not remain statistically significant after FDR correction; original *P* values are omitted for brevity and provided in Tables S3-S8 at the OSF repository [36]; demographic variables and group are included in models 2 to 9 as control variables, but their parameter estimates are omitted for brevity.

c β: standardized regression coefficient.

d FDR: false discovery rate.

e OR: odds ratio.

f HMP: Healthy Minds Program.

**Table 4.** Study 1: predictors of depression and anxiety symptom worsening^a^.

Model and predictor	Depression symptom worsening			Anxiety symptom worsening		
	OR^b^ (95% CI)		*P* value (FDR^c^)	OR (95% CI)		*P* value (FDR)
Model 1						
Age	1.15 (0.94-1.39)		.49	1.01 (0.81-1.22)		.90
Race: White	0.97 (0.44-2.33)		.95	0.80 (0.38-1.77)		.72
Gender: female	0.92 (0.43-2.07)		.94	1.31 (0.62-2.98)		.72
Group: spaced	1.72 (0.83-3.69)		.49	0.74 (0.36-1.47)		.72
Model 2						
Baseline depression	0.61 (0.42-0.87)		.08	0.91 (0.64-1.28)		.72
Model 3						
Baseline anxiety	0.92 (0.63-1.33)		.80	0.46 (0.31-0.66)		.001
Model 4						
Baseline loneliness	0.86 (0.59 -1.24)		.61	0.80 (0.56-1.13)		.71
Model 5						
Baseline experiential avoidance	1.03 (0.71-1.49)		.94	0.93 (0.66-1.32)		.72
Model 6						
Baseline fear of missing out	0.71 (0.47-1.04)		.45	1.11 (0.78-1.58)		.72
Model 7						
Baseline self-compassion	0.82 (0.56-1.17)		.49	0.92 (0.65-1.31)		.72
Model 8						
Minutes of HMP^d^ practice	0.90 (0.76-1.07)		.49	0.92 (0.77-1.11)		.72
Days of HMP usage	1.22 (0.88-1.77)		.49	1.35 (0.97-1.98)		.71
Model 9						
Baseline barriers to meditation	1.28 (0.88-1.87)		.49	0.89 (0.62-1.26)		.72

a Some estimates had CIs excluding the null value (OR 1) but did not remain statistically significant after FDR correction; original *P* values are omitted for brevity and provided in Tables S3–8 at the OSF repository [36]; demographic variables and group are included in models 2‐9 as control variables, but their parameter estimates are omitted for brevity.

b OR: odds ratio.

c FDR: false discovery rate.

d HMP: Healthy Minds Program.

#### Frequency of General AEx Occurrence

Higher baseline levels of depression (odds ratio [OR] 2.40, 95% CI 1.78‐3.33; *P_FDR_*<.001), anxiety (OR 2.13, 95% CI 1.61‐2.87; *P_FDR_*<.001), loneliness (OR 1.70, 95% CI 1.31‐2.24; *P_FDR_*<.001), and experiential avoidance (OR 2.49, 95% CI 1.85‐3.42; *P_FDR_*<.001) were significantly associated with a greater likelihood of reporting more frequent AExs. Self-compassion was related to a lower likelihood of AExs (OR 0.72, 95% CI 0.55‐0.93; *P_FDR_*=.02). Other variables did not predict more frequent AExs (*P_FDR_*>.05).

#### Reporting at Least One Specific MRAES-MBP AEx

The likelihood of experiencing any of the specific AExs was significantly associated with higher baseline depression (OR 1.47, 95% CI 1.16‐1.89; *P_FDR_*=.009), anxiety (OR 1.79, 95% CI 1.39‐2.35; *P_FDR_*<.001), loneliness (OR 1.39, 95% CI 1.09‐1.78; *P_FDR_*=.02), barriers to meditation (OR 1.42, 95% CI 1.11‐1.83; *P_FDR_*=.02), and experiential avoidance (OR 1.80, 95% CI 1.40‐2.34; *P_FDR_*<.001). Other predictors were not significant (*P_FDR_*>.35).

#### Specific MRAES-MBP AEx Count

In the zero‐inflated negative binomial analyses, we first examined the negative binomial count submodel and then the zero-inflation submodel (ie, prediction of excessive zeros). None of the variables predicted AEx count (*P_FDR_*>.08). In the zero-inflation submodel, baseline anxiety (OR 0.42, 95% CI 0.24‐0.73; *P_FDR_*=.02) and experiential avoidance (OR 0.43, 95% CI 0.23‐0.82; *P_FDR_*=.046) significantly predicted reporting zero specific AExs, such that a 1-SD increase in baseline anxiety was associated with a 58.1% lower odds (95% CI 26.8%‐76.1%) and a 1-SD increase in baseline experiential avoidance was associated with a 56.5% lower odds (95% CI 18.4%‐76.8%) of reporting zero specific AExs. Other predictors were nonsignificant (*P_FDR_*>.06).

#### Depression and Anxiety Symptom Worsening

Model fit improved significantly with the addition of baseline depression (*P*=.009) and anxiety (*P*<.001); however, only baseline anxiety was associated with a lower probability of anxiety symptom worsening (OR 0.87, 95% CI 0.31‐0.66; *P_FDR_*=.001), likely pointing to a ceiling effect (ie, individuals with high baseline anxiety had less room to show further increases). No other variables significantly predicted symptom worsening for either outcome (*P_FDR_*>.08).

### Discussion

Study 1 explored the prevalence, severity, and predictors of AExs among participants in a digital meditation training program. A total of 27.9% (88/315) of participants reported AExs that they attributed to meditation, and 6.7% (21/315) of participants reported some degree of functional impairment that usually lasted for 1 day or less. The study showed 10.3% attrition (35/351). Some participants may have dropped out because of AExs related to their use of the HMP. Under the most conservative assumption that all participants who dropped out experienced an AEx, the estimated prevalence would increase to 35.3% (124/351). Higher baseline depression, anxiety, loneliness, experiential avoidance, and perceived barriers to meditation were consistently associated with more AExs, whereas self-compassion showed a protective association in 1 model. Demographic characteristics and meditation dosage (eg, number of HMP usage days and meditation practice minutes) did not significantly predict AExs. These findings provided the basis for further confirmatory testing of identified predictors of AExs.

## Study 2

### Study 2 Overview

Study 2 drew data from a randomized trial using a 4-week HMP intervention in a distressed sample of US adults. This study aimed to estimate the prevalence of AExs in this sample and to test whether the AEx predictors identified in study 1 would replicate. On the basis of study 1 findings, we hypothesized that higher baseline depression, anxiety, loneliness, and perceived barriers to meditation would be associated with (1) more frequent AExs, (2) greater odds of reporting at least one specific AEx from the MRAES-MBP, and (3) a higher total number of reported specific AExs from the MRAES-MBP. Additionally, we exploratorily examined whether these same predictors were associated with depression and anxiety symptom worsening.

We made one deviation from the preregistration. We originally intended to conduct 2 sets of analyses on specific AExs (hypotheses 2 and 3), one including all reported AExs and another limited to those rated as “very distressing” or “somewhat distressing.” However, the number of participants who rated their experiences as distressing (n=73) was too small to support adequate model estimation. As a result, we do not report the second set of analyses. This deviation limited the study’s ability to identify risk factors for experiencing specific AExs from the MRAES-MBP that were subjectively evaluated as distressing; however, due to an insufficient number of participants reporting them, stable estimation was not possible.

We also conducted several exploratory analyses that were not preregistered. In study 2, participants’ compensation was not tied to their engagement with the HMP. Consequently, 7.8% of the sample (31/396) completed only the introductory guided meditation, which lasted approximately 5 minutes, and 4% (16/396) completed only the psychoeducation lessons without engaging in any guided meditations. This created a quasi-experimental setting that allowed us to compare AEx rates between participants who only received minimal or no exposure to meditation (and therefore were unlikely to have experienced AExs due to meditation) and those who were exposed to meditation.

Participants in study 1, including those who experienced AExs, generally reported being glad to have practiced meditation. Hence, in study 2, we aimed to isolate experiences that were perceived as unequivocally negative. To achieve this, as described in the following section, we removed the words “challenging” and “difficult” from the general AEx occurrence item (as experiences generally viewed as nonharmful may be challenging and difficult, eg, learning to play a musical instrument). We also included follow-up items assessing to what extent participants evaluated specific AExs that they experienced as distressing or positive. In addition, to avoid biasing participants by querying only negative experiences, we also assessed positive experiences that participants may attribute to their meditation practice.

### Methods

#### Ethical Considerations

All methods and procedures were reviewed and approved by the University of Wisconsin–Madison Institutional Review Board (protocol 2023‐1773) and carried out in accordance with the provisions of the World Medical Association Declaration of Helsinki. All participants provided their written informed consent electronically via REDCap before participating. Participants were eligible to receive up to US $125 if they completed surveys at all time points. Participants were able to opt out of the study at any time without penalty. Identifying information was collected for compensation purposes only and kept confidentially on a secure university server. The data used in this study were deidentified before sharing.

#### Participants and Procedure

Data collection occurred between June and December 2024. Community adults from all 50 US states were recruited through university-wide research recruitment emails at a large university in the Midwestern United States, via a research matching website, and through social media (eg, Craigslist). Inclusion criteria included age ≥18 years, elevated distress levels (*T*-scores ≥55 on the computer adaptive PROMIS Depression and/or PROMIS Anxiety [38]), proficiency in English, and access to a smartphone. Exclusion criteria included prior experience with meditation, defined in the same way as in study 1, prior use of the HMP app, history of psychosis or mania, the Alcohol Use Disorders Identification Test [53] (score ≥13 for women and ≥15 for men), and Drug Use Disorders Identification Test [54] (score ≥8 for women and men). The baseline sample was comprised of 530 participants; 396 (74.7%) of them completed the postintervention AEx measures, 417 (78.7%) completed depression and anxiety symptom measures, and 398 (75.1%) completed measures of positive experiences attributed to meditation. Participant demographics are presented in Table S1 at the OSF repository [36].

**Figure 4.**
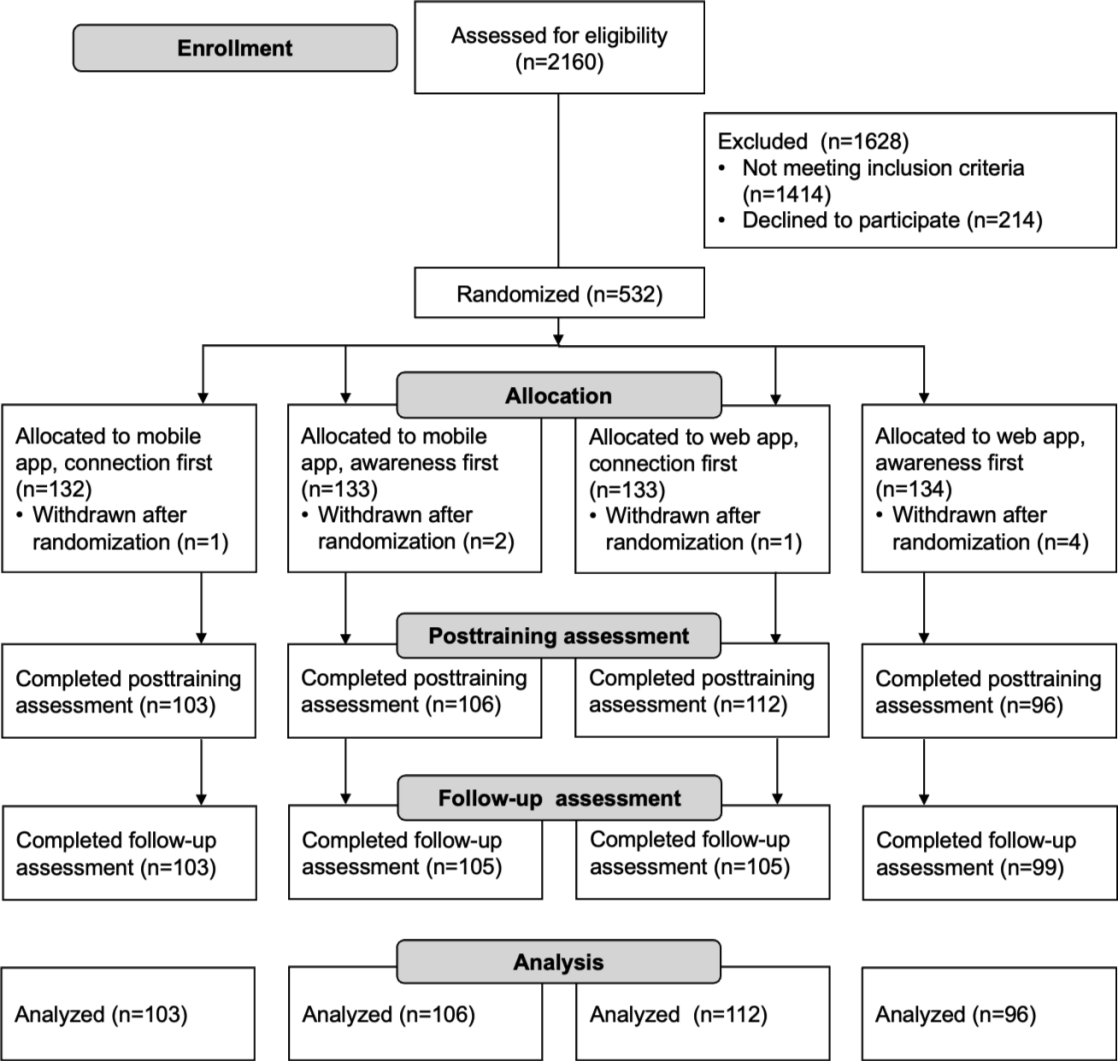
CONSORT (Consolidated Standards of Reporting Trials) diagram.

All participants enrolled in a 4-week HMP intervention. The primary aim of this trial was to compare different intervention delivery formats (smartphone app vs web app) and thematic sequences (awareness module first vs connection module first). Participants were randomized in equal proportions (1:1:1:1) to the 4 combinations of these conditions. Figure 4 presents the CONSORT diagram.

All participants received the same didactic content and guided meditation practices. In the current analyses, condition was included as a control variable. Data were collected at 6 time points: baseline assessment; weeks 1, 2, and 3 of the training; posttraining assessment (4 wk postbaseline); and follow-up assessment (4 mo postbaseline). This study uses data from the baseline and posttraining measurements. Unlike study 1, payment was not prorated according to HMP usage in study 2.

#### Measures: Predictors

Depression and anxiety were measured with the computer adaptive PROMIS Depression and Anxiety Scales, Item Bank version 1.0 [38], which yield *T*-scores as in study 1. Sample reliability statistics were not calculated for these measures because they use an algorithm that adapts to each participant’s responses, leading to different sets of items for different participants. In the validation study, the scales were evaluated using item response theory; test information functions indicated that the measures achieved measurement precision corresponding to a classical reliability of 0.95 [38]. Loneliness (sample α=0.92; ω=0.94), perceived barriers to meditation (sample α=0.77; *ω*=0.83), and HMP usage were measured with the same instruments as in study 1. Descriptive statistics are presented in Table S2 at the OSF repository [36].

#### Measures: Outcomes

AExs were assessed using the same measures as in study 1, with 3 exceptions. First, we modified the general AEx description from “challenging, difficult, or distressing experiences as a result of my meditation” to “distressing experiences that seemed to be caused by my use of the Healthy Minds application.” Second, we changed the item measuring participants’ evaluations of AExs from “I am glad I have practiced meditation” to “I am glad I have used the Healthy Minds application.” Third, to further probe participants’ evaluations of their experience, each specific AEx item from the MRAES-MBP was followed by the question, “What was this experience like for you?” with response options ranging from 1 (*very distressing*) to 5 (*very positive*). We exploratorily assessed positive experiences subjectively attributed to meditation using items that mirrored those used to measure AExs: “I had noticeably positive experiences that seemed to be caused by my use of the Healthy Minds application during this study” (1=*never* to 5=*frequently*); “My positive experiences made me more capable of functioning compared to what I’m used to” (1=*not at all* to 4=*severely*); and “How long did these positive effects last?” (1=*1 day or less* to 4=*longer than 1 mo*). We also asked participants about specific positive experiences that mirrored some of the specific AExs from the MRAES-MBP, using the prompt: “As a result of your use of the Healthy Minds application…” Items included “I felt relaxed or less stressed,” “I felt more connected to other people,” “My sleep seemed more refreshing,” and “I appreciated the people and things in my life more.”

#### Statistical Analysis

We conducted the analyses using the same statistical models as in study 1. As planned, FDR correction was applied to *P* values to account for multiple significance tests, following the same procedure as in study 1. As in study 1, we compared AEx rates between groups that did and did not experience depression and anxiety symptom improvement. We used the same statistical tests to compare AEx rates and proportions of participants experiencing depression and anxiety symptom worsening between meditating and nonmeditating groups.

#### Sample Size Justification

This study is a secondary analysis of data from a randomized trial that was originally powered to detect small between-group differences (*d*=0.20) on the primary outcome, as outlined in the parent trial preregistration [34]. A sensitivity analysis using the *pwr* R package [52] indicated that the available sample (n=396 participants who completed posttest AEx measures) provides 80% power to detect zero-order correlations of *r*≥0.14 between AEx predictors and AEx occurrence.

### Results

#### Prevalence of AExs

The distributions of AEx measures were similar to those in study 1 (see Figure S2 at the OSF repository [36]). The majority of participants (356/396, 89.9%) did not report any AExs attributed to their HMP use, 5.3% (91/396) reported experiencing them rarely, 3.5% (14/396) occasionally, and 1.3% (5/396) regularly or frequently. Of the 10.1% (40/396) who did report some AExs, the majority (28/396, 7.1%) reported no impairment, 2% of the full sample (8/396) indicated that AExs somewhat impaired their ability to function, while 0.5% (2/396) reported moderate impairment, and 0.3% (1/396) reported severe impairment. Among the 2.8% of participants who experienced impairment, 1.8% of the full sample (7/396) reported it lasting 1 day or less, 0.8% of the full sample (3/396) experienced it for a few days to 1 week, and 0.3% of the full sample (1/396) experienced it for more than 1 month. When asked about specific AExs from the MRAES-MBP, 41.9% (166/396) of participants reported experiencing at least one. On average, participants reported 0.84 AExs (SD 1.46). Similarly to study 1, the distribution was positively skewed. Anxiety and being bothered by little things were the most frequently reported AExs, while headaches or body pain were the least frequently reported. The distributions of specific AExs are shown in Figure S3 at the OSF repository [36].

Some participants experienced a clinically significant worsening of their depression (34/417, 11.8%) or anxiety symptoms (42/417, 10.1%) across 4 weeks of the study. During the same period, 55.2% (230/417) and 61.4% (256/417) of the participants experienced a clinically significant improvement of ≥3 *T*-score points decrease in depression and anxiety symptoms, respectively. None of the AEx measures differed between participants who did and did not experience improvement in depressive or anxiety symptoms (*P* values>.48).

Similarly to study 1, the majority of the participants indicated that they were glad to have used HMP, including 93.3% (332/356) of those who did not experience any AExs (ie, selected “never” on the AEx frequency item) and 90% (36/40) of those who did experience AExs (ie, selected any other response; see Figure S4 at the OSF repository [36]). Next, we asked participants who experienced an AEx from the MRAES-MBP [30] (n=166) to rate how they felt about each specific AEx they experienced during the study, using a scale ranging from 1 (*very distressing*) to 5 (*very positive*), with a neutral midpoint. A total of 44% (73/166) of these participants reported at least one distressing experience, and 56% (93/166) endorsed at least one neutral or positive experience; the rest of the sample did not report any of the listed experiences. Some experiences were rated as more distressing, such as feeling distant from people (mean 2.50, SD 1.10) and reexperiencing past stressful events (mean 2.51, SD=1.03). Others were viewed as more neutral, including trouble thinking (mean 3.06, SD=0.91) and being bothered by small things (mean 3.07, SD=0.93). A few were evaluated as more positive, such as increased sensitivity to sound (mean 4.10, SD 0.98). Distributions of evaluations for each specific AEx are shown in Figure S5 at the OSF repository [36].

#### Prevalence of Positive Experiences

**Figure 5.**
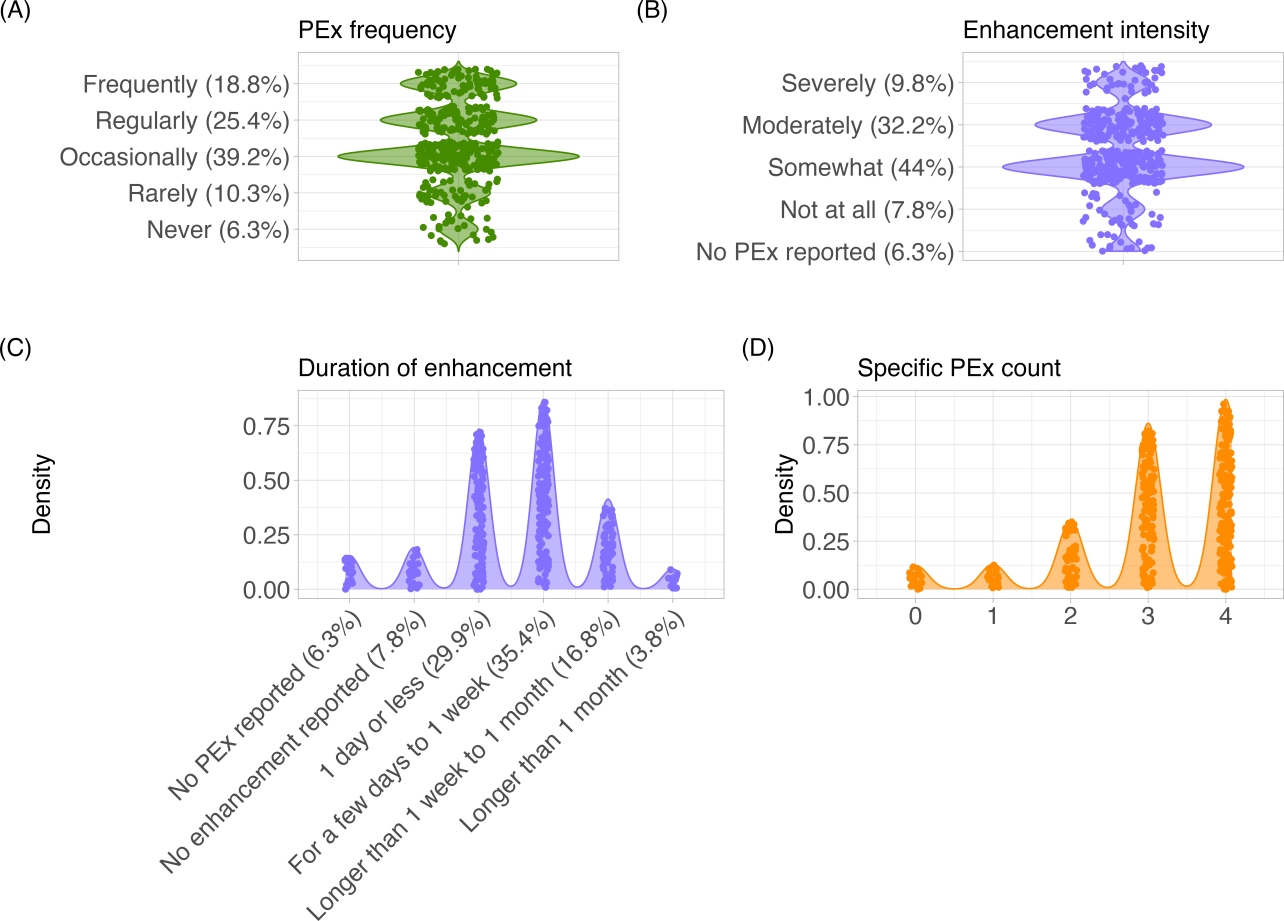
Prevalence of positive experiences (PEx). PEx frequency refers to how often participants had noticeably positive experiences that seemed to be caused by their use of the Healthy Minds app during this study; enhancement refers to improved ability to function; specific PEx count refers to the number of endorsed positive experiences from the items mirroring some of the items from the Meditation-Related Adverse Effects Scale, Mindfulness-Based Program version (out of 4) [30].

A large majority of the participants (373/398, 93.7%) reported positive experiences subjectively attributed to the use of HMP, and 85.9% (342/398) felt more capable of functioning (Figure 5). A total of 95% (378/398) of participants experienced at least one specific positive experience (eg, better sleep), and the majority reported 3 or all 4, as shown in Figure 5. On average, participants reported 3.0 (SD 1.1) positive experiences. The distributions of specific positive experiences are shown in Figure S6 at the OSF repository [36].

#### Predictors of AExs

Model estimates and their statistical significance are presented in Tables5^7^; full results are provided in Tables S9–14 at the OSF repository [36].

**Table 5.** Study 2: predictors of general adverse experience (AEx) count and reporting at least one specific MRAES-MBP^a^ AEx^b^.

Model and predictor	General AEx frequency		At least one specific MRAES-MBP AEx	
	OR^c^ (95% CI)	*P* value (FDR^d^)	OR (95% CI)	*P* value (FDR)
Model 1				
Age	1.01 (0.99‐1.04)	.43	1.02 (1.00‐1.03)	.20
Race: White	0.81 (0.41‐1.60)	.57	0.75 (0.50‐1.13)	.30
Gender: female	0.38 (0.19‐0.74)	.008	0.97 (0.63‐1.49)	.88
Group: app, connection first	1.83 (0.68‐5.21)	.29	1.08 (0.61‐1.91)	.88
Group: web, awareness first	1.22 (0.41‐3.65)	.72	1.18 (0.67‐2.10)	.72
Group: web, connection first	2.07 (0.82‐5.73)	.17	0.94 (0.54‐1.65)	.88
Model 2				
Baseline depression	1.41 (1.01‐1.99)	.06	1.16 (0.94‐1.43)	.30
Model 3				
Baseline anxiety	1.15 (0.82‐1.62)	.45	1.23 (1.00‐1.52)	.20
Model 4				
Baseline loneliness	1.54 (1.10‐2.18)	.02	1.10 (0.90‐1.35)	.47
Model 5				
Baseline barriers to meditation	1.47 (1.05‐2.09)	.04	1.21 (0.99‐1.50)	.20

a MRAES-MBP: Meditation-Related Adverse Effects Scale, Mindfulness-Based Program version [30].

b Some estimates had CIs excluding the null value (OR 1) but did not remain statistically significant after FDR correction; original *P* values are provided in Tables S3–8 at the OSF repository [36]; demographic variables and group are included in models 2‐9 as control variables, but their parameter estimates are omitted for brevity.

c OR: odds ratio.

d FDR: false discovery rate.

**Table 6.** Study 2. Predictors of MRAES-MBP^a^ adverse experience count^b^.

Model and predictor	Negative binomial submodel		Zero-inflation submodel	
Model 1	β^c^ (95% CI)	*P* value (FDR^d^)	OR^e^ (95% CI)	*P* value (FDR)
Age	0.00 (−0.01 to 0.02)	.94	0.81 (0.62 to 1.06)	.24
Race: White	−0.49 (−0.93 to −0.06)	.10	0.14 (0.00 to 44.49)	.70
Gender: female	−0.09 (−0.55 to 0.36)	.84	0.60 (0.04 to 9.42)	.84
Group: app, connection first	−0.48 (−0.95 to −0.02)	.13	–	–
Group: web, awareness first	−0.12 (−0.58 to 0.33)	.78	–	–
Group: web, connection first	0.04 (−0.40 to 0.47)	.94	–	–
Model 2				
Baseline depression	0.16 (−0.01 to 0.33)	.17	0.76 (0.35 to 1.62)	.70
Model 3				
Baseline anxiety	0.20 (0.02 to 0.38)	.20	0.97 (0.48 to 1.96)	.94
Model 4				
Baseline loneliness	0.30 (0.13 to 0.46)	.006	1.40 (0.66 to 2.99)	.61
Model 5				
Baseline barriers to meditation	0.26 (0.09 to 0.42)	.01	0.44 (0.09 to 2.06)	.52

a MRAES-MBP: Meditation-Related Adverse Effects Scale, Mindfulness-Based Program version [30].

b Some estimates had CIs excluding the null value (β=0) but did not remain statistically significant after FDR correction; original *P* values are provided in Tables S3–8 at the OSF repository [36]; demographic variables and group are included in models 2‐9 as control variables, but their parameter estimates are omitted for brevity; group was removed from the zero-inflation submodel for MRAES-MBP AEx count due to weak identification and unstable estimates.

c β: standardized regression coefficient.

d FDR: false discovery rate.

e OR: odds ratio.

**Table 7.** Study 2: predictors of depression and anxiety symptom worsening^a^.

Model and predictor	Depression symptom worsening		Anxiety symptom worsening	
	OR^b^ (95% CI)	*P* value (FDR^c^)	OR (95% CI)	*P* value (FDR)
Model 1				
Age	0.99 (0.97‐1.02)	.72	0.99 (0.96‐1.01)	.48
Race: White	0.70 (0.37‐1.29)	.72	0.62 (0.31‐1.21)	.33
Gender: female	1.27 (0.66‐2.55)	.72	1.01 (0.51‐2.06)	.98
Group: app, connection first	1.12 (0.47‐2.71)	.86	1.53 (0.56‐4.38)	.53
Group: web, awareness first	1.51 (0.65‐3.59)	.72	2.04 (0.78‐5.75)	.33
Group: web, connection first	1.03 (0.43‐2.49)	.95	1.82 (0.71‐5.05)	.35
Model 2				
Baseline depression	0.64 (0.46‐0.89)	.06	0.78 (0.56‐1.09)	.33
Model 3				
Baseline anxiety	0.87 (0.65‐1.18)	.72	0.62 (0.44‐0.85)	.03
Model 4				
Baseline loneliness	0.95 (0.71‐1.28)	.86	0.97 (0.71‐1.33)	.93
Model 5				
Baseline barriers to meditation	0.89 (0.66‐1.21)	.72	1.25 (0.90‐1.74)	.33

a Some estimates had CIs excluding the null value (OR 1) but did not remain statistically significant after FDR correction; original *P* values are provided in Tables S3–8 at the OSF repository [36]; demographic variables and group are included in models 2‐9 as control variables, but their parameter estimates are omitted for brevity.

b OR: odds ratio.

c FDR: false discovery rate.

#### Frequency of AEx Occurrence

In the demographic-only baseline model, gender was the only significant predictor of AEx frequency: women had lower odds compared to other genders (OR 0.38, 95% CI 0.19‐0.74; *P_FDR_*=.008). Loneliness (OR 1.54, 95% CI 1.10‐2.18; *P_FDR_*=.02) and perceived barriers to meditation (OR 1.47, 95% CI 1.05‐2.09; *P_FDR_*=.04) were significant in subsequent models. Other predictors were nonsignificant (*P_FDR_*>.06).

#### Reporting at Least One Specific MRAES-MBP AEx

Adding baseline anxiety significantly improved model fit over the baseline model (*P*=.045); however, none of the predictors were significant after FDR correction (*P_FDR_*>.20).

#### Specific MRAES-MBP AEx Count

Higher baseline loneliness (β=.30, 95% CI 0.13‐0.46; *P_FDR_*=.006) and more perceived barriers to meditation (β=.26, 95% CI 0.09‐0.42; *P_FDR_*=.01) were associated with reporting 35.7% (95% CI 13.6%‐59.1%) and 28.6% more specific AExs (95% CI 9.6%‐52.1%), respectively. None of the variables were significant in the zero-inflation submodel (*P* values*_FDR_*>.24).

#### Depression and Anxiety Symptom Worsening

In the series of models for the probability of depression symptom worsening, only the addition of baseline depression significantly improved model fit (*P*=.007); however, none of the predictors remained significant after FDR correction (*P* values*_FDR_*>.06). Adding baseline anxiety significantly improved the model for anxiety symptom worsening (*P*=.003), and higher baseline anxiety was associated with lower odds of symptom worsening (OR 0.62, 95% CI 0.44‐0.85; *P_FDR_*=.03), suggesting a ceiling effect.

#### AExs in Meditating and Nonmeditating Groups

There were no differences in AExs between meditating and nonmeditating groups with respect to the average AEx frequency (*r*=−0.69; *P*=.63), the proportion of participants reporting at least one specific AEx from the MRAES-MBP (*h*=0.09; *P*=.59), the average count of specific AExs from the MRAES-MBP (*r*=−0.69; *P*=.81), or depression (*h*=−0.18, *P*=.18) and anxiety symptom worsening (*h*=−0.19; *P*=.17). Descriptive statistics and full results of statistical tests are presented in Table S15 at the OSF repository [36].

### Discussion

Study 2, a preregistered confirmatory follow-up to study 1, found a lower overall prevalence of AExs that participants attributed to meditation, with 10.10% (40/396) of participants reporting experiencing AExs compared to 27.9% (88/315) in study 1. In this study, attrition was 25.3% (134/530). Assuming most conservatively that all participants who dropped out experienced an AEx, the estimated prevalence would increase to 32.8% (174/530), which is comparable to the estimate in study 1 (124/351, 35.3%). Impairment was reported by 3% (12/396; 21/315, 6.7%, in study 1). This difference could be attributed to emphasizing the distressing nature of these experiences in study 2 (ie, removing “challenging” and “difficult” from the item), lower baseline anxiety in study 2 sample (sample 1: mean 63.97, SD 5.59; sample 2: mean 61.54, SD 6.27), and differences in the populations sampled (ie, undergraduates vs general population adults). Compared with study 1, fewer participants in study 2 reported impairment or clinically significant symptom worsening, with 90% (36/40) of the participants who had distressing experiences indicating that they were still glad to have used HMP and 56% (93/166) evaluating at least one of the experiences from the MRAES-MBP as neutral or positive. Furthermore, 93.7% (373/398) of the participants reported deriving positive experiences from their engagement with HMP, suggesting predominantly positive appraisals relative to the 10.1% AEx rate. In terms of predictors, study 2 confirmed that higher baseline loneliness and perceived barriers to meditation were significantly associated with more AExs. However, contrary to study 1, baseline anxiety and depression were not consistent predictors of AExs. A protective effect of female gender and White race emerged in study 2, whereas demographic variables had no predictive utility in study 1. The prevalence of AExs and symptom worsening did not differ between participants who did and did not complete guided meditations, showing that at least some of the reported AExs stemmed from sources other than meditation. Overall, study 2 corroborated several findings of study 1 while yielding a lower estimate of AEx prevalence and providing a more nuanced picture of AEx genesis and participants’ subjective evaluations of their experiences.

## Study 3

### Methods

Study 1 included an open-ended question, “What was helpful in responding to/managing challenging, difficult, or distressing meditation-related experiences?” in the postintervention survey. We analyzed participants’ responses using qualitative document analysis [55], a flexible, inductive method for identifying patterns of meaning (themes) from the data. This approach maximizes the preservation of participants’ voices by allowing themes to emerge from the data in a “bottom-up” manner rather than imposing predefined categories. Data analysis was conducted by 3 coders trained in qualitative coding. Through an iterative process of initial coding, data segments were systematically labeled until consensus among coders was achieved. This was followed by axial coding to identify overarching themes and relationships between labels [56]. Once consensus was reached, a narrative analysis of the themes was conducted. Pseudonyms were used to maintain participants’ confidentiality.

### Results

Responses varied from a few words to several sentences; the majority were brief. We identified 8 core themes summarizing the strategies that participants used to cope with AExs. Several participants described seeking social support (theme 1) from family, friends, or mental health professionals. For example, Avery reported, “talking to a therapist about my frustrations.” Others reported connecting to a broader sense of humanity through feelings of connection (theme 2), such as warmth, oneness, or an invisible bond with others. For example, Alex found it “helpful to remind myself I was not alone” and that “many people go through similar difficult experiences and they will be there for me.” Parker shared, “I went and wished well on others until I felt better about myself.”

Some participants coped by stepping away from the practice (theme 3), either mentally or physically. Sam shared, “I would take time away from the practice to find something to soothe my mind,” while Casey said they would “get up and complete a different task.” Others responded by accepting the present moment (theme 4), staying present with nonjudgment. Casey reflected on “reminding myself to acknowledge the thought and not judge it but that it’s okay if the mind wanders,” while Kai shared, “trying to ground myself or be present in the moment.” This acceptance often led to re-engaging with the practice (theme 5) through strategies such as refocusing attention, following the instructor’s guidance, or trying a different meditation technique. Casey noted that “slowly bringing [the mind] back to the breath was helpful.” Justice shared: “I used other practices that were taught in the previous week to try and get over that.”

Participants also sought to express and understand their emotions (theme 6) through introspection, crying, or talking with others. Jordan explained this as “[remaining] calm and thinking through why I was feeling the emotions I was feeling.” In the same vein, Dakota engaged in “reflecting on why I was feeling any challenge or distress.” To manage overwhelm (theme 7), participants reported slowing down the pace of the program, relaxing, using breathwork, taking breaks, spending time alone, or using medication. Taylor said they would “take some time to calm down and unwind,” while Ari noted they would “pause the practice and wait until I was ready to meditate a small amount of time later.”

Finally, participants engaged in reflective and reframing techniques (theme 8), such as prioritizing personal needs, adopting a growth mindset, or practicing gratitude and compassion. Quinn described using positive reframing: “Appreciate the difficulties and challenges I have faced and move forward with a willingness to be better.” Similarly, Phoenix used positive self-talk to cope: “primarily I had to remind myself that I am important too.”

### Discussion

The qualitative findings provided context for the quantitative results, offering insight into how participants understood and navigated AExs that emerged during the study. Participants described a wide range of coping strategies, highlighting the diversity of ways individuals relate to and manage challenging experiences. Many drew on skills learned through the HMP or from prior experience. These responses emphasized participants’ psychological resilience and capacity for self-regulation in the face of discomfort. As one participant noted, “while facing these experiences can be difficult, it’s nice to remember that facing them can improve your life overall.”

## General Discussion

### Principal Findings

In this series of studies, we investigated the prevalence and predictors of self-reported AExs and worsening symptoms that occurred during participants’ engagement with the HMP, a self-guided digital MBI, as well as participants’ subjective evaluations of their experiences and coping strategies. The prevalence estimates were largely consistent with prior literature. Higher baseline depression, anxiety, loneliness, experiential avoidance, and perceived barriers to meditation predicted more AExs. Most participants evaluated learning to meditate positively, including those who reported AExs, and described using a variety of coping strategies, including techniques learned through HMP.

The 27.9% (88/315) of participants prospectively evaluated prevalence of self-reported AExs that participants attributed to meditation observed in study 1 aligned with estimates from previous survey studies that used the same measure in a retrospective observational study context (32%) [11,12]. Study 2 found a lower prevalence of 10.1% (40/396). This discrepancy may be partially due to the wording of the item in study 2, which specifically emphasized distressing effects, rather than more broadly including “challenging” or “difficult” experiences that some participants may actually perceive as neutral or positive. For example, learning to regulate attention through meditation practice is likely challenging or difficult for most individuals. However, lower prevalence rates were also observed across other AEx measures, suggesting that participants in study 2 experienced fewer AExs overall, possibly due to lower baseline symptoms. Pooled across the 2 studies, the prevalence of AExs attributed to meditation was 18% (128/711). The proportion of participants endorsing at least one item on the MRAES-MBP [30] was 65.4% (206/315) in study 1 and 41.9% (166/396) in study 2 (pooled 52.3%, 372/711). This pattern is consistent with prior research indicating that checklist-based self-report assessments yield higher AExs prevalence estimates compared to single-item or open-ended questions [6,11,12]. Across both studies, 10 (1.4%) of the 711 participants reported AExs occurring regularly or frequently. Eight (1.1%) participants reported moderate to severe impairment attributed to meditation, and 4 (0.6%) reported impairment lasting longer than 1 week.

In study 1, 10.8% (34/316) and 12% (38/316) of participants and in study 2, 11.8% (49/417) and 10.1% (42/417) experienced a clinically significant ≥3 *T*-score increase of depression and anxiety symptoms, respectively (pooled 11.3%, 83/733, for depression and 10.9%, 80/733, for anxiety). This estimate is higher than the estimated 3.6% rate of clinically significant symptom worsening among participants of the Mindfulness-Based Stress Reduction program offered by a community health clinic, an estimate calculated from participants’ records collected over 14 years (n=2155) [29]. Baer et al [13] reported symptom deterioration rates (2%‐6%) in an RCT similar to community Mindfulness-Based Stress Reduction. One explanation for the discrepancy is that this study applied a lower threshold for clinically significant symptom worsening. In our samples, the MCID of 3 *T*-score points [45,46] corresponds to roughly 0.4 SD on PROMIS Depression and 0.5 *SD* on PROMIS Anxiety. In contrast, Baer et al [13] used a higher cutoff—approximately 1 SD on their depression and anxiety measures. Hirshberg et al [29] defined clinically significant harm as a shift from functional to moderate, or from moderate to severe symptom levels, an approach that generally requires a larger change in symptoms than the MCID used here. To contextualize these findings, the share of the participants who experienced depression and anxiety symptom improvement was 51.9% (164/316) and 45.6% (144/316), respectively, in study 1, and 55.2% (230/417) and 61.4% (256/417), respectively, in study 2 (pooled 53.8%, 394/733 for depression and 54.6%, 400/733 for anxiety).

Across both studies, baseline loneliness consistently predicted AExs, suggesting that individuals who felt more isolated were more vulnerable to difficult experiences. This was further supported by findings from the qualitative study 3, in which some participants described using their actual or perceived social connections as a way to cope with AExs. Those with higher baseline loneliness may have had less social support or sense of connection. Thus, social context may serve as both a risk factor and a protective buffer in the occurrence of and coping with AExs.

In study 1, baseline levels of depression and anxiety were associated with several AEx outcomes; however, this was not replicated in study 2. Baseline experiential avoidance emerged as a risk factor across several AEx measures in study 1; however, it was not assessed in study 2. This finding was supported by the qualitative data, where some participants described coping with challenging experiences by expressing and processing their emotions. Individuals with a stronger tendency to avoid unwanted internal experiences may have been less able or willing to engage in this form of coping, potentially increasing their vulnerability to AExs.

Neither the number of days using the app nor total meditation minutes predicted AExs in Study 1. This contrasts with Britton et al [6], who found evidence that AExs emerge according to the principle of biological gradient, where greater exposure leads to a higher incidence of the experience. However, the Britton et al study involved in-person mindfulness-based cognitive therapy with much higher exposure—an average of over 32 hours of formal and informal practice during the 8-week program and approximately an additional 26 hours of practice until the 3-month follow-up, when AExs were evaluated. In our study, participants averaged 3.76 hours of meditation (SD 1.99) during the 2-week study 1 period, and 1.27 hours of meditation (SD 1.53) during the 4-week study 2 period. Notably, perceived barriers to meditation predicted AExs in both studies, suggesting that subjective anticipated difficulties with the practice, such as internal resistance, frustration, or misunderstanding, may contribute to negative outcomes.

Importantly, in study 2, participants who, as determined by the objective app logs, had minimal (<5 min) or no exposure to guided meditations reported AExs they attributed to using the HMP app at the same rate as those who engaged in guided meditations. This suggests that these experiences were not caused by meditation and therefore cannot be classified as AEfs. When considered alongside the above results that baseline symptoms and participant characteristics seem to be the most robust predictors of AExs, at least among the variables we assessed, our data provide a caution against the practice of inferring causality from self-attribution or observational data. Although the temporality criterion is satisfied in such data—experiences occurred during or following meditation practice—the specificity criterion cannot be evaluated without a comparison condition, as emphasized in the Bradford Hill framework for causal inference [57]. Prior work has similarly found that participants sometimes attribute experiences to MBIs that are better explained by unrelated causes or natural symptom fluctuations [25].

Most participants evaluated their meditation experience and use of HMP positively, including 89.8% (79/88) of those who reported AExs in study 1 and 90% (36/40) in study 2. This estimate is similar to Baer et al [13], who reported that between 85% and 92% of the participants who had AExs rated them as not at all upsetting or only somewhat upsetting. An observational study [11] also found that 88% of the survey participants who had AExs were still glad to have practiced meditation. Future research could further disentangle the immediate valence and appraisal of experiences during meditation training from the interpretive meaning-making that practitioners construct around those experiences.

In sum, the contemplative science field has made substantial strides in recent years in documenting negative experiences that may arise during meditation training [8]. Our study extended this work by examining such experiences in the context of a digital MBI. The overall low rates of reported AExs and impairment, the absence of statistically significant differences between meditating and nonmeditating groups, the high proportion of participants who experienced an AEx yet still endorsed being glad to have meditated, and the resilience reflected in participants’ coping strategies collectively argue against generalized concerns about potential widespread harms associated with the digital MBI evaluated here. That said, a small number of participants may report serious or prolonged AExs during digital meditation training. Such experiences should be monitored in both research and clinical contexts, and their causal connection to the intervention should be systematically evaluated.

### Clinical Implications

This study holds important public health implications because meditation apps are the most widely used category of mental health mobile apps, with millions of active users [15,16]. US Food and Drug Administration guidelines require the collection and reporting of adverse events in sponsored trials of investigational devices; however, because the vast majority of digital meditation interventions are classified as wellness devices and are therefore outside the scope of US Food and Drug Administration–cleared digital therapeutics, the onus remains on the creators to monitor for challenging experiences [58,59]. Across digital mental health apps broadly, participants report AExs such as symptom worsening, distress caused by app features, self-harm or suicidal ideation or intent, and rehospitalization, with frequencies ranging from common to very rare, and differing widely based on the clinical population targeted with the app [20]. Given the frequency with which AExs occur, their screening and reporting should be integrated into digital mindfulness programs and apps. Our data also suggest that meditation programs should inform new practitioners that, as in daily life, distressing experiences may occur during meditation. Having realistic expectations about the kinds of experiences that can arise during meditation will allow individuals to make informed choices about participating in meditation training. In addition, some researchers have conjectured that when novice practitioners encounter challenging experiences, they may be more likely to discontinue practice as a result [12]. If meditation programs more fully inform individuals about the range of potential experiences and that some degree of distress during meditation is not uncommon and should even be expected for most participants [6,13], new practitioners may be better able to work with such challenges.

In the data we reviewed, the majority of reported AExs were brief and short-lived. However, a subset of individuals may experience more intense and longer-lasting AExs that they subjectively attribute to meditation. Developers of meditation programs are encouraged to incorporate screening for vulnerability factors—such as those identified in this and other studies—into their offerings. Individuals who are flagged as high risk for AExs could be more closely monitored through in-app questionnaires and, when clinically indicated, escalated to receive support within the app, be directed to external resources for meditators experiencing distress, or given information on general mental health support (eg, calling an emergency crisis line).

It is clear from prior qualitative research that some AExs attributed to meditation [10,60], particularly intensive meditation practice and/or esoteric practice of the sort not encountered in secular meditation programs or apps, are more severe, long-lasting, and potentially debilitating. Future research on meditation AExs and AEfs would benefit from differentiating between types of meditation practice, in terms of techniques, duration, intensity, and context (eg, app vs in person; with a trained teacher vs without a trained teacher). Indeed, many meditation traditions speak of the potential negative consequences of meditation practiced with the wrong understanding, or in the wrong context, or without an appropriate teacher [61].

### Research Implications

The research makes a few contributions to the field. First, we emphasize the importance of distinguishing between AExs—any distressing experiences occurring during an intervention—and AEfs, which refer to harmful experiences that are *caused* by the MBI and would not otherwise occur. As these data illustrate, participants report meditation-related AExs even in the absence of meditation. Hence, subjective attribution alone cannot be taken as sufficient evidence of causality. Rare events, such as severe or prolonged AExs and their possible relationship to meditation, may be studied on a case-by-case basis, as previously suggested [6]. However, we encourage the researchers to rely on established causal inference methods (eg, RCTs) to evaluate whether more common mild AExs should be classified as AEfs—that is, iatrogenic effects caused by meditation. Beyond RCTs, hybrid designs such as stepped-wedge trials could evaluate AExs in real-world app deployments.

Second, given the prevalence of distressing or difficult experiences in the context of meditation, it is important for future research to actively monitor and report AExs in clinical trials, with particular attention to how they are operationalized. Our study yielded varying AEx prevalence estimates depending on the measures used. Therefore, the way AExs and AEfs are defined and measured plays a critical role in shaping findings. Researchers are encouraged to clearly specify how AExs and AEfs are defined in their studies, which measurement scales are used, the degree to which their study design allows causal inferences to be drawn, and to interpret their findings in light of those methodological choices.

### Limitations and Future Directions

There are several limitations to note. First, although the study advanced prior observational research on AExs in digital MBIs by establishing temporal precedence and using standardized meditation training, it did not include a control group. As a result, we were unable to compare AExs in meditation training to a base rate and test the causal relationship between meditation and AExs. Second, while attrition rates in this study (study 1: 36/351, 10.3% and study 2: 134/530, 25.3%) were at or below estimates for attrition in RCTs of mental health apps generally [62], it is possible that some participants who discontinued their involvement in our studies did so due to AExs related to their use of the HMP. This could lead to an underestimate of AExs based on those who completed postintervention measures. Under the most conservative assumption that all participants who dropped out experienced an AEx, the estimated prevalence would increase to 35.3% (124/351) in study 1 and 32.8% (174/530) in study 2. Third, these data are useful for understanding AExs in the context of digital trainings in which participants received relatively low doses of meditation—an average of 3.76 hours in study 1 and 1.27 hours in study 2. We cannot infer from these data the impacts of digital or in-person meditation training when the dose is 10 times or, in very intensive contexts, 50 times greater. Future trials could explicitly contrast app-based secular practices with more intensive forms of practice (eg, standardized 8-wk MBIs and meditation retreats). Additionally, although using several different operationalizations and measures allowed us to capture a broad range of AExs, the study relied exclusively on self-report measures. Previous studies have reported objective adverse events, such as hospitalization occurring during the trial [63]; however, such severe adverse events are rare. To better evaluate mild AExs of the type reported here, future studies could adapt AEx measures for informant report and combine self-reported and informant-reported AExs to enable more objective assessment. Finally, this study mainly focused on the risk factors associated with AExs, but it may be illuminating for research to more closely examine protective factors.

## Abbreviations

AEf: adverse effect
AEx: adverse experience
CONSORT: Consolidated Standards of Reporting Trials
FDR: false discovery rate
HMP: Healthy Minds Program
MBI: mindfulness-based intervention
MCID: minimal clinically important difference
MRAES-MBP: Meditation-Related Adverse Effects Scale for Mindfulness-Based Programs
OR: odds ratio
OSF: Open Science Framework
PROMIS: Patient-Reported Outcomes Measurement Information System
RCT: randomized controlled trial
REDCap: Research Electronic Data Capture
